# NPI-0052 and γ-radiation induce a synergistic apoptotic effect in medulloblastoma

**DOI:** 10.1038/s41419-019-2026-y

**Published:** 2019-10-16

**Authors:** Eleni Frisira, Fatima Rashid, Swastina Nath Varma, Sara Badodi, Valentine Ayodele Benjamin-Ombo, David Michod, Maria Victoria Niklison-Chirou

**Affiliations:** 1 Blizard Institute, Barts and the London School of Medicine and Dentistry, Queen Mary University of London, 4 Newark Street, London, E1 2AT UK; 2 Institute of Child Health, University College of London, London, WC1N 1EH UK

**Keywords:** CNS cancer, Paediatric cancer

## Abstract

Medulloblastoma (MB) is the most common malignant solid paediatric brain tumour. The standard treatment for MB is surgical resection of the tumour, radiation and chemotherapy. This therapy is associated with high morbidity and adverse side effects. Hence, more targeted and less toxic therapies are vitally needed to improve the quality of life of survivors. NPI-0052 is a novel proteasome inhibitor that irreversibly binds the 20S proteasome subunit. This compound has anti-tumour activity in metastatic solid tumours, glioblastoma and multiple myeloma with a good safety profile. Importantly, NPI-0052 has a lipophilic structure and can penetrate the blood–brain barrier, making it a suitable treatment for brain tumours. In the present study, we performed an in silico gene expression analysis to evaluate the proteasome subunit expression in MB. To evaluate the anticancer activity of NPI-0052, we used a range of MB patient-derived MB cells and cell lines. The synergistic cell death of NPI-0052 with γ-radiation was evaluated in tumour organoids derived from patient-derived MB cells. We show that high expression of proteasome subunits is a poor prognostic factor for MB patients. Also, our preclinical work demonstrated that NPI-0052 can inhibit proteasome activity and activate apoptosis in MB cells. Moreover, we observe that NPI-0052 has a synergistic apoptotic effect with γ-radiation, a component of the current MB therapy. Here, we present compelling preclinical evidence that NPI-0052 can be used as an adjuvant treatment for p53-family-expressing MB tumours.

## Introduction

Medulloblastomas (MBs) are the most common primary malignant brain tumours of childhood^1^. Survival of these children has improved in the past decades due to aggressive surgical resection, followed by high-dose craniospinal irradiation and chemotherapy. This treatment approach has come at a significant cost for survivors, with the majority of patients suffering from significant long-term neurocognitive, endocrine and other toxicities^2^. Furthermore, recurrence is common and often proves fatal^3^. An adjuvant therapy holds the promise of offering a more effective and less toxic treatment for children with MB.

Historically, MB has been classified into four molecular subgroups, namely: Wingless (good prognosis), Sonic Hedgehog (intermediate prognosis), Group 3 (G3—worst prognosis) and Group 4 (G4—intermediate prognosis)^4^. New reports reveal that the four MB subgroups can be classified into 12 subgroups highlighting the complex genetic landscape of these tumours^5^. G3-MB and G4-MB are the most aggressive and least characterized of all subgroups. It predominantly occurs in young children and is associated with MYC or MYCN amplification; however, p53 is never mutated^3^. Importantly, we recently reported that G3-MB and G4-MB express high levels of p73, a member of the p53 family^6^. In these tumours, p73 sustains cell proliferation by activating glutamine metabolism.

The ubiquitin proteasome pathway (UPP) is the principal mechanism for protein catabolism in human cells^7^. The UPP complex is in charge of the degradation of intracellular proteins. These include damaged proteins and those that must be maintained at low concentrations such as pro-apoptotic proteins like the p53 family. Therefore, the UPP plays a central role in the regulation of apoptosis, cell cycle and cell proliferation^8^. The first step in the UPP process involves the marking of proteins with ubiquitin molecules. Targeted proteins are recognized by the 26S proteasome complex, composed of the catalytic 20S core subunit and the 19S regulator subunits^9^. Increased proteasome activity has been demonstrated in several human tumours, suggesting that tumour cells are more sensitive to proteasome inhibitors than normal cells^10^. Proteasome inhibitors are drugs that either reversibly or irreversibly block the activity of the 26S proteasome complex. NPI-0052, also known as Marizomib or Salinosporamide A, is a second-generation proteasome inhibitor that binds irreversibly to the 20S core subunit of the 26S proteasome^11^. NPI-0052 is in phase I/II trials conducted in patients with solid tumours, lymphoma, glioblastoma and relapsed and/or refractory multiple myeloma. Notably, NPI-0052 does not demonstrate toxicity to normal cells at pharmacological concentrations^12^ and is able to penetrate the blood–brain barrier. Indeed, it has been shown to inhibit the proteasome activity of glioblastoma brain tumours, with minimal cytotoxic effects on normal human neural stem cells^13^. In most cases, tumour cell death resulting after proteasome inhibitor treatment requires caspase activation and has been linked to increased levels of reactive oxygen species (ROS)^14^.

In this study, we sought to investigate the effect of NPI-0052 in patient-derived G3/G4-MB and MB cell lines. First, we show that MB tumours express high levels of the 26S proteasome complex, which is a poor prognostic factor for MB patients. We also demonstrate that NPI-0052 induces inhibition of proteasome activity with subsequent apoptosis activation in MB cells. Interestingly, we show that the p53 family plays a substantial role in NPI-0052’s mechanism of action by increasing ROS levels. Notably, NPI-0052 has a synergistic effect with γ-radiation, a component of the current MB therapy. These findings raise the possibility that NPI-0052 can be used as an adjuvant treatment for the most aggressive and invasive MB tumours.

## Material and methods

### Cell culture

MB cells, DAOY and ICb-1299, were cultured at 37 °C in humidified 5% CO_2_ in Dulbecco’s modified Eagle’s medium (DMEM) + GlutaMAX medium (Gibco), supplemented with 10% (v/v) foetal bovine serum (FBS, Gibco) and penicillin/streptomycin (1 U/mL, Gibco), as previously described^15^.

CHLA-01-MED (ATCC^®^ CRL-3021) and CHLA-01R-MED (ATCC^®^ CRL-3034) were cultured 37 °C in humidified 5% CO_2_ in DMEM:F12 medium (Gibco) with 20 ng/mL human epidermal growth factor, 20 ng/mL human basic fibroblast growth factor and 2% (v/v) B-27 supplement (Invitrogen, cat. no.17504).

DAOY cells were purchased from ATCC (ATCC^®^ HTB186™). The primary human MB cells, ICb-1299, were obtained from Dr. Xiao-Nan Li (Baylor College of Medicine, Texas Children’s Cancer Centre, USA). The human primary MB cells CHLA-01-MED and CHLA-01R-MED were donated by Prof. Silvia Marino (Queen Mary University of London, UK).

### NPI-0052 preparation

NPI-0052 (AdipoGen^®^) was dissolved in dimethyl sulfoxide (DMSO). All controls for each experiment were treated with DMSO with equal volumes as the drug treatment for the rest of the conditions.

### Cell irradiation

Cells and organoids were irradiated with γ-radiation using a caesium (^137^Cs) irradiator at the dose rate of 2 or 4 gray (Gy) per minute at UCL-Institute of Child Health^16^.

### Knockdown of p73

Small interfering RNA (siRNA) constructs targeting p73 (sip73*1: ID: 2671; sip73*2: ID: 115666) and a non-targeting control siRNA (scramble) were purchased from Ambion. Cells were transfected with 10 pM siRNA with Lipofectamine 3000 (Life Technologies) according to the supplier’s protocol.

### Apoptosis determination

Apoptosis was quantified using the Annexin V-FITC Determination Kit (eBioscience) according to the manufacturer’s instructions.

### Cell viability

Cell viability was quantified by CellTiter-Glo^®^ Luminescent Cell Viability Assay according to the manufacturer’s instructions.

### Measurement of total glutathione and GSH/GSSG levels

MB cells were plated and treated as indicated. Reduced and oxidized glutathione (GSSG) ratio was measured by using the glutathione (GSH)/GSSG-Glo Assay Kit (Promega) according to the manufacturer’s protocol.

### Mitochondrial membrane potential

3,3′-Dihexyloxacarbocyanine iodide (Invitrogen, cat. no. D273) staining was carried out as previously described^6^.

### Hydrogen peroxide assay

Amplex Red Hydrogen Peroxide/Peroxidase Assay Kit (Thermo Fisher Scientific) according to manufacturer’s specification.

### Tumour organoids

Tumour organoids were grown as previously described^17^ with some modifications. Two hundred microlitres of tumour cell suspension (1 × 10^6^ cells) was mixed with 800 μL of cold Matrigel (Life Sciences). Twenty microlitres of this suspension was pipetted onto a dimpled parafilm mould. Once the Matrigel droplets had set after 15 min incubation at 37 °C, they were transferred to a 10 cm tissue culture dish and suspended in 10 mL of the appropriate medium. Tumour organoids were incubated at 37 °C in humidified CO_2_ without shaking for 4 days. Then, the tumour organoids were transferred to an orbital shaker and cultured at 70 revolutions per minute (r.p.m.). The medium was changed twice weekly.

### Immunostaining

Paraffin-embedded tumour organoids were sectioned at 3 μm and staining for haematoxylin and eosin (H&E), p53 (Dako, prediluted), cleaved caspase-3 (Novocastra, prediluted) and γ-H_2_AX (H2A histone family member X) (Roche, prediluted) were performed by UCL IQPath (Institute of Neurology, London, UK).

### Image capturing and analysis

Histological slides were digitized on a LEICA SCN400 scanner (Leica UK) at ×40 magnification and 65% image compression setting.

### Statistical analysis

All results are expressed as mean values ± SD or mean values ±SEM of at least three independent experiments, except when otherwise indicated. The unpaired Student’s *t* test and analysis of variance (one-way ANOVA) were used to compare and identify statistically significant differences. Statistically significance levels were represented as *P < 0.05, **P < 0.01 and ***P < 0.001.

## Results

### High proteasome subunits levels identify MBs with poor overall survival rate

**Fig. 1:**
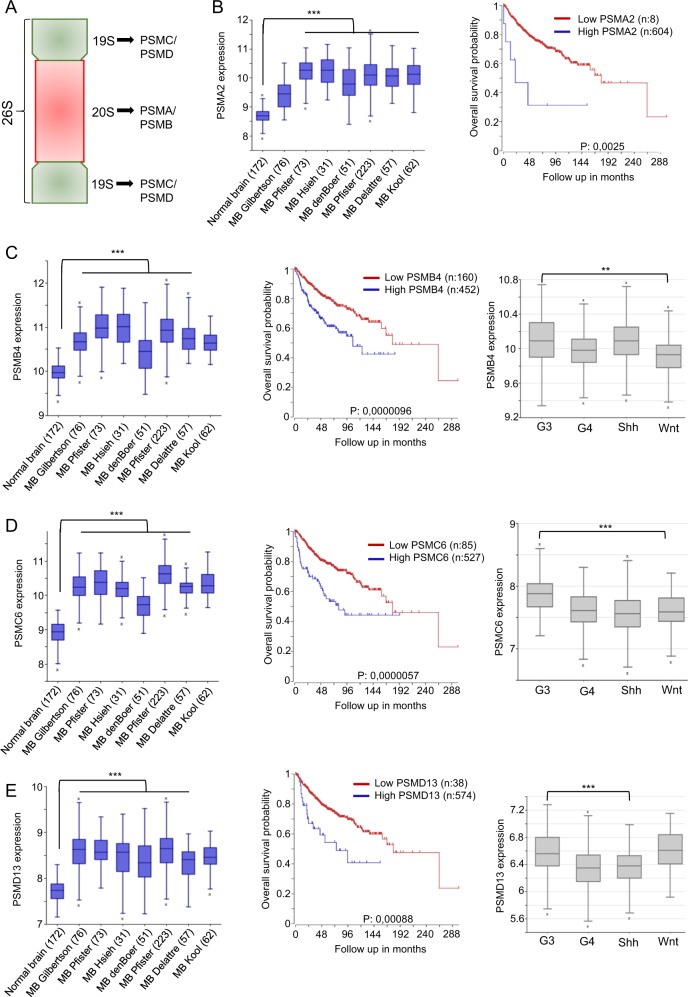
26S overexpression indicates poor prognosis in MB patients. **a** Diagram of the 26S proteasome complex. The 26S proteasome is formed by two subcomplexes: 19S (regulatory complex) and 20S (core proteasome). **b** Expression of PSMA2 in normal brain (172) vs. human MB tumours. Kaplan–Meier survival curve based on high and low PSMA2 expression levels in MB tumours derived from Cavalli cohort with 763 patients. Tumours with high PSMA2 expression showed decreased survival (*P* = 0.0025). **c** PSMB4 in normal brain (172) vs. human MB tumours. Kaplan–Meier survival curve based on high and low PSMB4 expression levels in MB tumours derived from Cavalli cohort with 763 patients. Tumours with high PSMB4 expression showed decreased survival (*P* = 0.0000096). PSMB4 levels in the four different groups: Group 3, Group 4, Wingless (WNT) and Sonic Hedgehog (SHH). The data are derived from Cavalli cohort with 763 patients (**P = 0.003). **d** PSMC6 in normal brain (172) vs. human MB tumours. Kaplan–Meier survival curve based on high and low PSMC6 expression levels in MB tumours derived from Cavalli cohort with 763 patients. Tumours with high PSMC6 expression showed decreased survival (*P* = 0.0000057). PSMC6 levels in the four different groups: Group 3, Group 4, WNT and SHH. The data are derived from Cavalli cohort with 763 patients (***P = 0.0001). **e** PSMD13 in normal brain (172) vs. human MB tumours. Kaplan–Meier survival curve based on high and low PSMD13 expression levels in MB tumours derived from Cavalli cohort with 763 patients. Tumours with high PSMD13 expression showed decreased survival (*P* = 0.00088). PSMD13 levels in the four different groups: Group 3, Group 4, WNT and SHH. The data are derived from Cavalli cohort with 763 patients (**P = 0.0001). Data were acquired from the R2: Genomics Analysis and Visualization Platform (https://hgserver1.amc.nl/cgi-bin/r2/main.cgi) and significance is calculated with a one-way ANOVA between groups

Little is known on the role of the proteasome in MB tumours. Therefore, to evaluate its clinical significance, we compared expression levels of different 19S and 20S proteasome subunits (Fig. 1a) in MB compared to normal brain samples using the R2 platform (www.r2.amc.nl). We found a substantial upregulation of all 19S (PSMD and PSMC) and 20S (PSMA and PSMB) proteasome subunits tested in MB (Fig. 1b–e, Fig. S1A). Importantly, Kaplan–Meier survival analysis demonstrated that patients with high levels of PSMA2, PSMA7, PSMB3, PSMB4, PSMC6 and PSMD13 expression had a significantly reduced overall survival as compared to MB patients with low expression levels (Cavalli data set, n:763, Fig. 1b–e, Fig. S1B). Next, we examined the subgroup-specific expression levels of the 19S (PSMD and PSMC) and 20S (PSMA and PSMB) proteasome subunits. Significant upregulation of PSMB1, PSMB4, PSMC2, PSMC6 and PSMD13 was found in the G3-MB (Fig. 1c–e, Fig. S1C).

Overall, our results demonstrate that the most aggressive subgroup of MB express high levels of the 26S proteasome by increased synthesis of the different proteasome subunits. This reflects a potential therapeutic target for MB tumours.

### NPI-0052 inhibits cell proliferation and induces apoptosis of G3/G4-MB cells in vitro

Next, we studied the ability of NPI-0052 to block the UPP in a range of patient-derived primary MB cells and cell lines. G3/G4 patient-derived MB lines, named ICb-1299, CHLA-01-MED and CHLA-01R-MED, were chosen to further dissect the role of NPI-0052 in the most aggressive and less characterized MB subgroups.

**Fig. 2:**
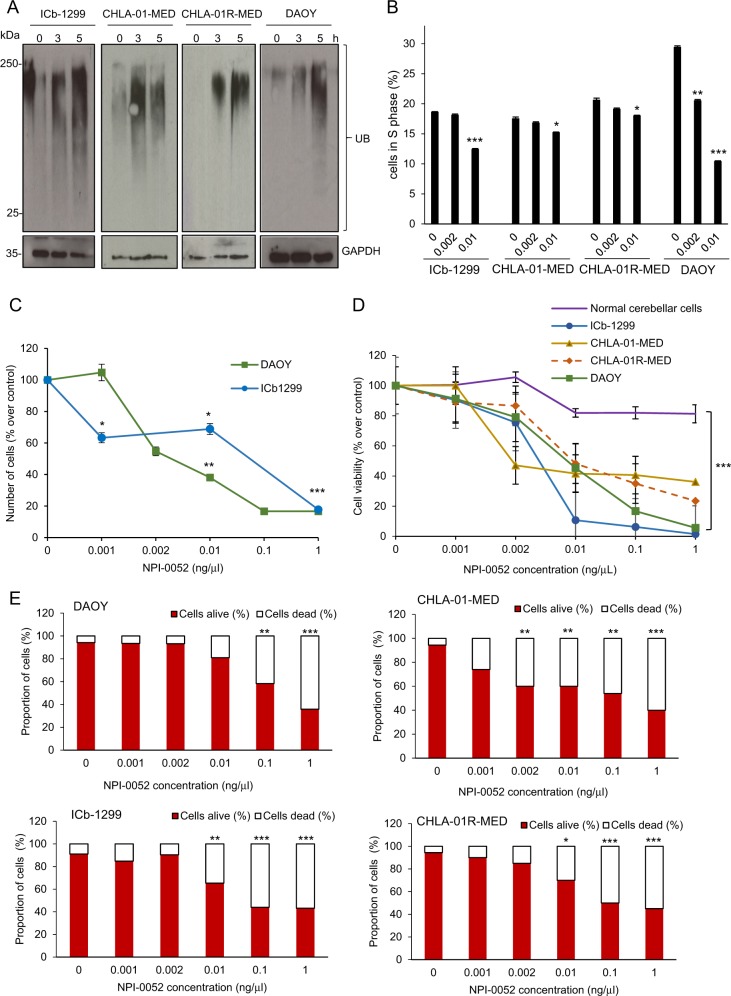
NPI-0052 inhibited proteasome activity and induced apoptosis of patient-derived G3/G4-MB cells. **a** Primary human MB cells (ICb-1299, CHLA-01-MED and CHLA-01R-MED) and medulloblastoma cell line DAOY were treated with 0.002 ng/μL NPI-0052 for 1, 3 and 5 h. Western blot against ubiquitinated proteins (UB) was performed. GAPDH was used as a loading control. **b** Quantification of the S phase of the cell cycle. MB cells (ICb-1299, CHLA-01-MED, CHLA-01R-MED and DAOY) were treated with NPI-0052 (0, 0.002 and 0.01 ng/μL) and after 18 h were fixed with 70% ethanol at 4 °C, stained for 30 min at room temperature with a PI-Triton-RNase PBS solution, and analysed on a FACS-Calibur flow cytometer. Statistical significance of the reduction in the cell number in S phase was calculated using the paired Student’s *t* test. **c**, **d** Human MB cells (ICb-1299, CHLA-01-MED, CHLA-01R-MED and DAOY) and normal post-mitotic cerebellar cells were treated with different concentrations of NPI-0052 (0, 0.001, 0.002, 0.01, 0.1 and 1 ng/μL). After 24 h the cells were collected. **c** Cell number was determined using a NucleoCounter^®^ NC-100™ (Chemometec) (*n* = 3); data are represented as mean ± SD. *P < 0.01; **P < 0.001; ***P < 0.0001. **d** Cell viability were determined with CellTiter-Glo (*n* = 4) ± SEM; ***P < 0.0001. **e** MB cells (ICb-1299, CHLA-01-MED, CHLA-01R-MED and DAOY) were treated with different concentrations of NPI-0052 (0, 0.001, 0.002, 0.01, 0.1 and 1 ng/μL). After 24 h cells were collected and apoptosis was measured with Annexin V-FITC and PI for flow cytometry analysis. Cells that stain negative for Annexin V-FITC and negative for PI were consider as “alive”. Dead cells were considered to be the apoptotic, necrotic and dead cells (*n* = 3). Data are represented as mean ± SD. *P < 0.01; **P < 0.001; ***P < 0.0001

First, we confirmed accumulation of ubiquitin-tagged proteins as a smear of proteins of different molecular weight on a western blot. We found that NPI-0052 induced accumulation of ubiquitinated proteins after 3 h of treatment in ICb-1299, CHLA-01-MED and CHLA-01R-MED (primary human G3/G4-MB) and in the DAOY cell line (Fig. 2a). These data suggest that NPI-0052 blocks proteasome activity in G3/G4-MB primary cells and cell lines.

It has been reported that proteasome inhibitors cause accumulation of the tumour suppressor proteins such as p53 and p73, which are crucial for cell cycle regulation^16,18^. Therefore, we performed a cell cycle analyses of MB cells after treatment with NPI-0052 using flow cytometry. We observed that after 24 h of NPI-0052 treatment, all MB cells became arrested in the S phase (Fig. 2b, Fig. S2A). This result indicates that the MB cells stop cell proliferation after NPI-0052 treatment, possibly due to DNA damage or replicative stress.

To validate this result, we measured the cell number after 24 h of NPI-0052 treatment. Importantly, we confirmed a significant reduction in ICb-1299 and DAOY cell number with increasing concentrations of NPI-0052 (Fig. 2c). Moreover, we detected a significant reduction in cell viability and an increase in apoptosis after 24 h of NPI-0052 treatment in a concentration-dependent manner (Fig. 2d, e, Fig. S2B, C). Since MB is a cerebellar tumour, we isolated granular cerebellar cells from postnatal mice and used them as a control to measure the toxicity of NPI-0052 in the post-mitotic cell. Notably, cell viability of normal cerebellar cells was not affected after 24 h of treatment with NPI-0052 (Fig. 2d).

Importantly, we observed that increasing the incubation time to 48 h induced a strong reduction in cell viability and increased apoptosis of MB cells after adding NPI-0052 (Fig. S2C, D).

Together, these data indicate that NPI-0052 is able to inhibit the 26S proteasome, repressing cell proliferation and inducing apoptosis in the most aggressive MB subgroups.

### NPI-0052 induces mitochondrial malfunction with ROS generation

**Fig. 3:**
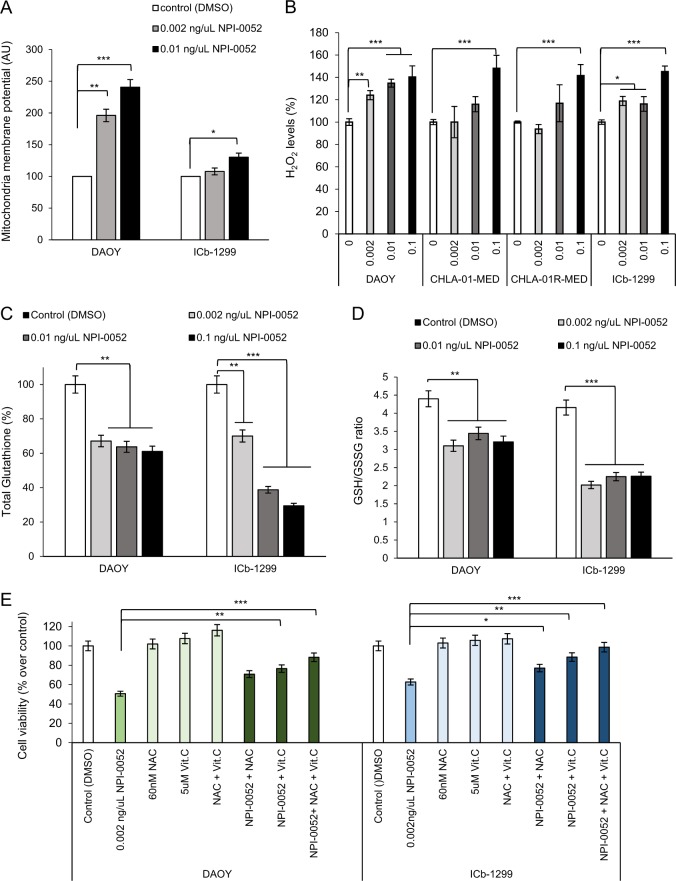
NPI-0052 induced oxidative stress in medulloblastoma cells. **a** MB cells (ICb-1299 and DAOY) were treated with 0, 0.002 and 0.01 ng/μL NPI-0052 for 24 h. MB cells were labelled with DiOC6(3) (3,3′-dihexyloxacarbocyanine iodide) a marker of mitochondrial membrane potential. Cells were analysed by flow cytometry and the geometric means were recorded (*n* = 3). Data are represented as geometric means ± SD. Values significantly higher than the control (*P < 0.01; **P < 0.001; ***P < 0.0001) are indicated. **b** MB cells (ICb-1299, CHLA-01-MED, CHLA-01R-MED and DAOY) were treated with 0, 0.002, 0.01 and 0.1 ng/μL of NPI-0052. Level of H_2_O_2_ generated after 18 h of treatment was measured using the Amplex red hydrogen peroxide assay (*n* = 3). Data are represented as mean ± SD. *P < 0.01; **P < 0.001; ***P < 0.0001. **c**, **d** DAOY and ICb-1299 cells were treated with different concentrations of NPI-0052 (0, 0.002 and 0.01 ng/µL) for 24 h. **c** Total glutathione and **d** reduced and oxidized glutathione ratio (GSH/GSSG) were measured by using the GSH/GSSG-Glo Assay Kit (*n* = 3). Data are represented as mean ± SD. **P < 0.001; ***P < 0.0001. **e** Human MB cells (ICb-1299 and DAOY) were treated for 18 h with 0.002 ng/μL NPI-0052, 60 nM *N*-acetyl-cysteine (NAC) or 5 μM vitamin C (Vit.C). Cell viability was determined with CellTiter-Glo (*n* = 3) ± SEM; *P < 0.01, **P < 0.001; ***P < 0.0001

It has been reported that some proteasome inhibitors induce cell death through oxidative stress caused by mitochondrial dysfunction^19^. Therefore, we assessed whether NPI-0052 induces mitochondrial hyperpolarization in MB cells. Significant mitochondrial hyperpolarization was observed after 18 h of NPI-0052 treatment in DAOY and ICb-1299 cells (Fig. 3a). Because mitochondrial hyperpolarization has been related to ROS production^19^, we measured hydrogen peroxide levels after 18 h of NPI-0052 treatment (Fig. 3b). Indeed, we detected a significant increase in hydrogen peroxide generation after NPI-0052 treatment in a concentration-dependent manner (Fig. 3b). To confirm these results, we determined the redox status of MB cells upon NPI-0052 treatment as assessed by the total GSH levels and ratio of reduced GSH to oxidized glutathione (GSSG) [GSH:GSSG] as an index of oxidative stress (Fig. 3c, d). We observed a substantial reduction in total GSH levels (Fig. 3c) with decreased ratio of reduced GSH to glutathione disulfide after NPI-0052 treatment (Fig. 3d). These findings verify that NPI-0052 induces oxidative stress in G3/G4-MB cells. Importantly, pre-treatment of MB cells with a combination of antioxidants, *N*-acetyl-cysteine (NAC) and vitamin C, rescued the reduction in cell viability observed after NPI-0052 treatment (Fig. 3e).

Taken together these data suggest that NPI-0052 is able to induce apoptosis by mitochondria dependent-ROS generation in the most aggressive MB subgroups.

### The p53-family members are key mediators of NPI-0052 pathogenesis in G3/G4-MB cells

**Fig. 4:**
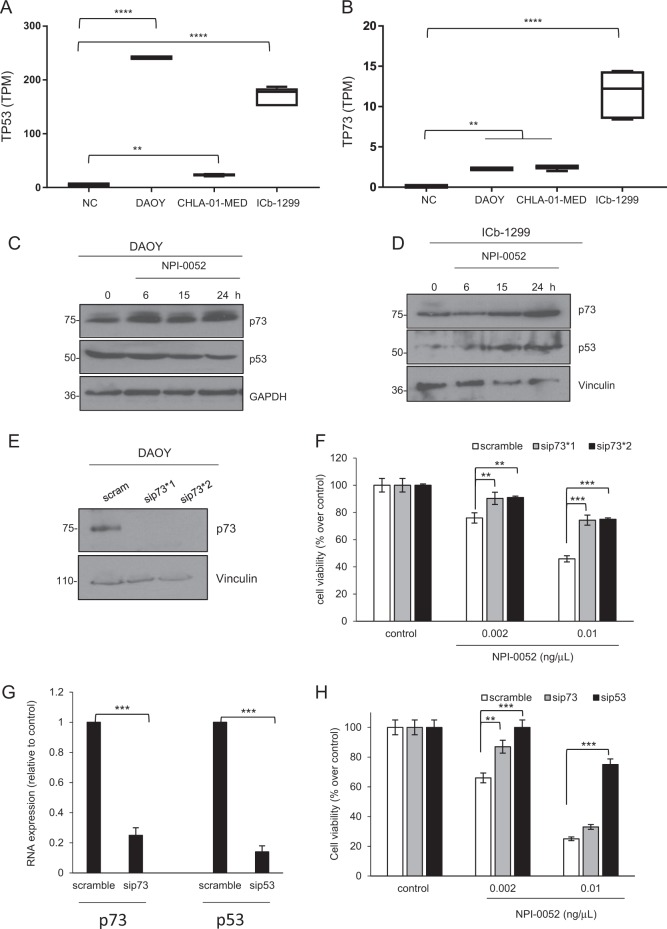
NPI-0052 induced stabilization of the p53 family. **a**, **b** Box plot representation of TP53 (**a**) and TP73 (**b**) expression levels (TPM: transcript per million). NC: normal cerebellum. **P < 0.001; ****P   < 0.00001. **c**, **d** Human MB cells DAOY (**c**) and ICb-1299 (**d**) were treated with 0.002 ng/μL NPI-0052 for 0, 6, 15 and 24 h. Cells were collected and a western blot of lysed cells probed with a primary anti-p73 (1/1000), anti-p53 (1/1000), GAPDH (1/5000) or vinculin (1/5000) antibodies. **e**, **f** DAOY cells were treated with two siRNA against p73 (sip73*1, sip73*2) for 48 h. **e** Cells were collected and a western blot of lysed cells for p73 and vinculin was performed. **f** Cell viability analysis in scramble, sip73*1 and sip73*2 DAOY cells after treatment with 0.002 or 0.01 ng/μL NPI-0052 for 24 h. **P  < 0.001; ***P  < 0.0001. **g**, **h** ICb-1299 cells were treated with a siRNA against p73 or p53 for 48 h. **g** Cells were collected and a RT-PCR for p73 and p53 was performed. ***P  < 0.0001 **h** Cell viability analysis in scramble, sip73 and sip53 ICb-1299 cells after treatment with 0.002 or 0.01 ng/μL NPI-0052 for 24 h. **P  < 0.001; ***P < 0.0001

To clarify the molecular mechanism mediating apoptosis in MBs after NPI-0052 treatment, we setup the following experiments. We assessed the involvement of p53 and p73 proteins, two members of the p53 family that are important in MB pathogenesis^3,6^. Extensive literature has also confirmed that the p53 family plays a significant role in inducing apoptosis after oxidative stress^18,20^. First, we assessed p53 and p73 RNA levels in our G3/G4-MB cells and MB cell line (CHLA-01-MED, ICb-1299, DAOY) compared to normal cerebellar cells. Higher expression of p53 and p73 was observed in all MB cells vs. control (Fig. 4a, b). Next, we treated DAOY and ICb-1299 cells with 0.002 ng/mL NPI-0052 for 6, 12 and 24 h. Western blot analysis demonstrated significant p73 stabilization in DAOY cells after NPI-0052 treatment, with no changes in p53 levels (Fig. 4c, Fig. S3A). This is in line with the DAOY cell line expressing a non-functional p53 protein^21^. We also observed substantial p53 and p73 stabilization after NPI-0052 treatment in ICb-1299 primary cells, which has p53-wild type^21^ (Fig. 4d, Fig. S3B).

Next, we set out to assess the impact of p53 or p73 silencing on MB cells. Indeed, knockdown of p73 in DAOY cells (Fig. 4e, f) or knockdown of p53 or p73 in ICb-1299 primary cells (Fig. 4g, h) significantly rescues the cell viability after NPI-0052 treatment.

In conclusion, these data highlight the central role of the p53 family in mediating cell death after NPI-0052 treatment in G3/G4-MBs, which normally express p53 and p73 proteins.

### Synergistic effect of NPI-0052 with radiation in MB cells expressing p53/p73 wild type

Current treatment for MB involves surgical removal of the tumour, followed by craniospinal (brain and spine) radiation and chemotherapy^2,22^. Combination treatment of radiation/chemotherapy with proteasome inhibitors could help reduce the therapeutic doses, thereby reducing their side effects.

**Fig. 5:**
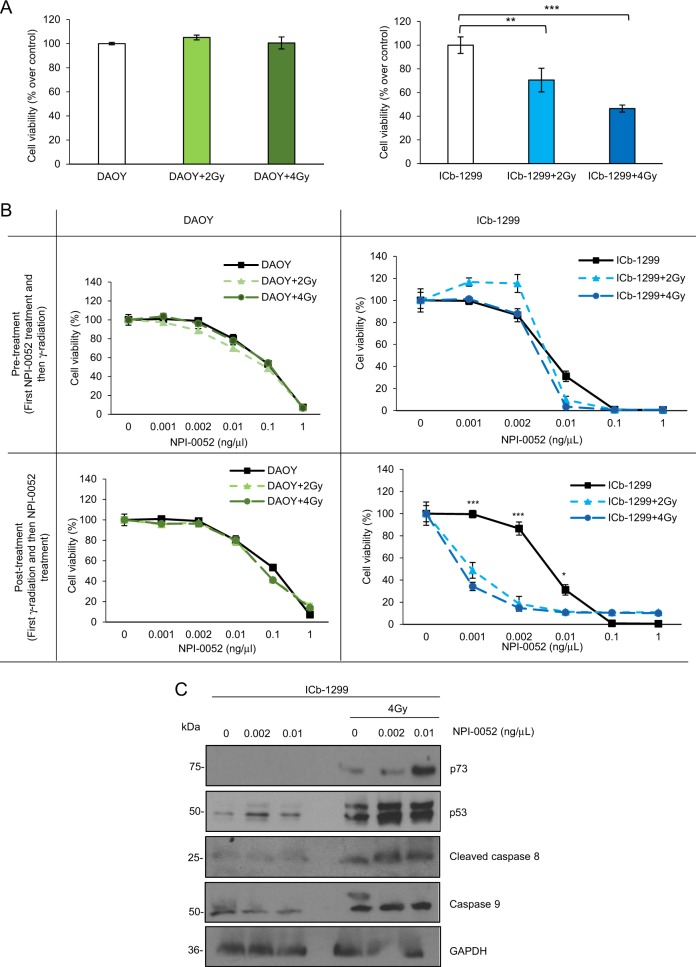
NPI-0052 plus γ-radiation induces a synergistic apoptotic effect in G3/G4-MB cells. **a** DAOY and ICb-1299 cells were treated with 0, 2 and 4Gy γ-radiation. After 24 h cell viability was measured with CellTiter-Glo (*n* = 4) ± SEM; **P < 0.001; ***P < 0.0001. **b** Dose–response curves fitted to experimental data for cell viability in DAOY and ICb-1299 treated with different concentrations of NPI-0052 alone or in combination with γ-radiation. Pre-treatment condition: MB cells were treated with different concentrations of NPI-0052 for 4 h, followed by treatment of 2 or 4Gy γ-radiation for 24 h. For the post-treatment condition: MB cells were treated with 2 or 4 Gy radiation doses, and 4 h later with different concentrations of NPI-0052 for 24 h. Cell viability was then measured with CellTiter-Glo (*n* = 3) ± SEM; *P < 0.01; ***P < 0.0001. **c** ICb-1299 cells were treated or not with 4 Gy γ-radiation for 4 h, followed by 0, 0.002 or 0.01 ng/μL NPI-0052. After 24 h cells were collected and a western blot of lysed cells was performed and probed with primary anti-p73 (1/1000), anti-p53 (1/1000), cleaved caspase-8 (1/1000), caspase-9 (1/1000) and GAPDH (1/5000) antibodies

Therefore, we first challenged DAOY and ICb-1299 cells with 2 or 4 Gy γ-radiation. Cell viability was assessed after 18 h of treatment. Figure 5a shows that a single dose of radiation as low as 2 or 4 Gy reduced ICb-1299 viability by around 30% and 70%, respectively. Importantly, no effect was observed in DAOY cells (Fig. 5a). This difference in sensitivities is in accordance with previous findings showing that TP53 mutant cells (like DAOY) exhibit superior radiation resistance compared to cells expressing p53 wild type^23^.

Next, we questioned whether NPI-0052 could induce a synergistic apoptotic effect with γ-radiation. To this end, we setup two different experiments. In one experiment, MB cells were treated with NPI-0052 first, followed by γ-radiation after 4 h (pre-treatment). In the second experiment, MB cells were treated with γ-radiation, followed by treatment with NPI-0052 4 h later (post-treatment). After 24 h, cell viability was assessed. No synergistic effect was seen in DAOY or ICb-1299 cells in the pre-treatment conditions (Fig. 5b). Importantly, we observed a significant synergistic effect between γ-radiation and NPI-0052 in ICb-1299 human primary cells in the post-treatment condition (Fig. 5b). However, no synergic effect was observed in DAOY cells (Fig. 5b).

Radiation therapy kills cancer cells by damaging their DNA and DNA damage-induced p53 stabilization^16,24^. To validate this hypothesis, we measured, by western blot, p53 and p73 levels in ICb-1299 human primary cells in the presence of different concentrations of NPI-0052 or combination treatment with 4 Gy and NPI-0052 treatment alone. We observed that the combination treatment of NPI-0052 plus radiation induced significant p53 and p73 stabilization (Fig. 5c). We also saw a clear increase in the levels of cleaved caspase-8 and caspase-9, two markers of apoptosis activation (Fig. 5c).

Together, these results support the hypothesis that G3/G4-MB primary cells that express wild-type p53 and p73 will show a synergistic effect between radiation and NPI-0052.

### Synergistic effect between NPI-0052 and radiation in organoids from human G3/G4-MBs

**Fig. 6:**
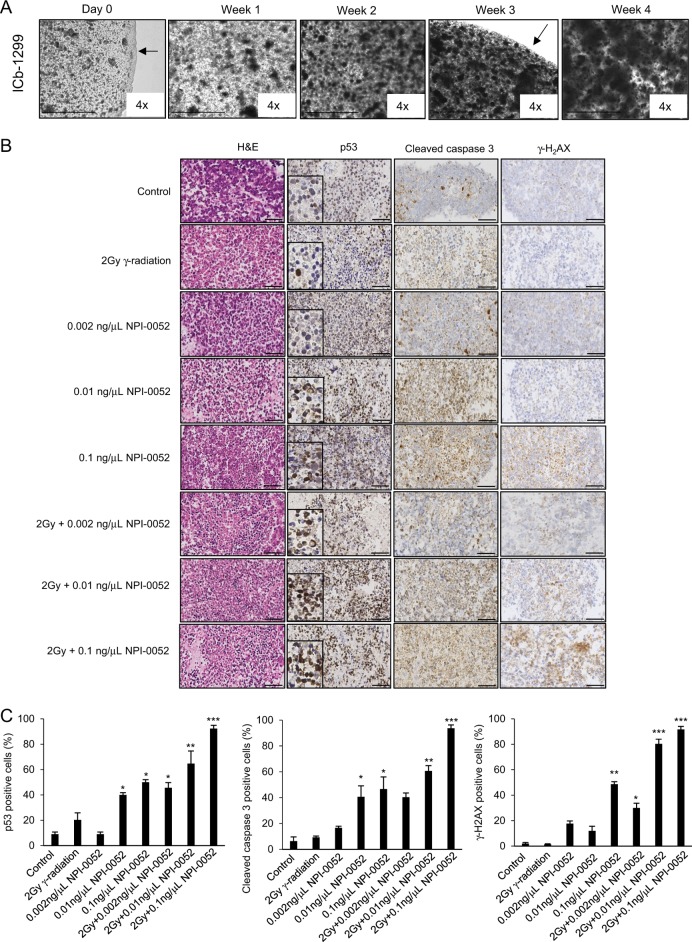
Organoid- ICb-1299 cells recapitulate the synergic apoptotic effect between γ-radiation and NPI-0052. **a** Establishment of organoid culture for ICb-1299 cells. Representative low-power (×4) microscope bright-field images of organoid cultures at indicated time points. Scale bar, 1000 μm. Arrowhead indicates edge of Matrigel droplet. **b**, **c** Histology of the ICb-1299 organoid treated with 2 Gy γ-radiation alone or different concentrations of NPI-0052 alone or 4 h treatment of 2 Gy γ radiation, followed by NPI-0052 treatment for 72 h. **b** Representative bright-field images are shown for haematoxylin and eosin (H&E), p53, cleaved caspase-3 and γ-H_2_AX. Scale bar, 50 µm. **c** Quantification is shown as the mean of positive cells per high-power field. *P < 0.01; **P < 0.001; ***P < 0.0001

To evaluate the relevance of these findings, we created tumour organoids from ICb-1299 primary cells. Organoids were grown over 4 weeks (Fig. 6a) and were then treated with or without 2 Gy radiation, followed by NPI-0052 treatment (Fig. 6b). All organoids had the morphological characteristics of MBs, as assessed by H&E stain (Fig. 6b). Furthermore, histopathological analysis revealed that increased concentrations of NPI-0052 induced p53 stabilization and cleaved caspase-3 expression (Fig. 6b). This indicates that NPI-0052 alone is sufficient to induce apoptosis activation. Importantly, this effect occurred in a concentration-dependent manner (Fig. 6c). Additionally, only high doses of NPI-0052 induced significant increase of γ-H_2_AX expression, an early marker of DNA damage (Fig. 6b). These results indicate that high doses of NPI-0052 induced significant ROS-mediated DNA damage in the MB organoids (Fig. 6c).

Moreover, we found that 2 Gy radiation followed by NPI-0052 significantly enhanced p53 stabilization, cleaved caspase-3 and γ-H_2_AX expression levels (Fig. 6c), suggesting that the combination treatment induced a synergic apoptotic effect in the organoids.

Together, these data support the conclusion that NPI-0052 treatment enhances radiation effect in the most aggressive MB subgroups via DNA damage and p53-induced apoptosis.

## Discussion

G3-MB and G4-MB are the most aggressive MB subgroups and often present with metastases at diagnosis^25^. Therefore, treatment is usually deemed to be aggressive too. However, associated adverse effects of chosen regimens often outweigh the possible benefits in a large proportion of patients^26^. Therefore, we decided to study the role of NPI-0052, a proteasome inhibitor, in MB to obtain proof of principle to develop a less aggressive G3-MB and G4-MB treatment.

We show here that 26S proteasome subunits are overexpressed in MB as compared to the normal cerebellum. Moreover, high levels of the 26S proteasome subunits identify a group of patients with a very poor outcome. These results suggest that MB tumours can be more sensitive to treatment with proteasome inhibitors than normal brain cells, a finding that confirms and further extends the original description that proteasome inhibitors act on different tumours with little effect on normal cells^10,13^.

However, this anti-tumoural effect of NPI-0052 has not been investigated in MB so far. Therefore, we used different G3/G4-MB patient-derived cells and a human cell line to demonstrate that NPI-0052 induces inhibition of 26S proteasome activity, followed by cell death in a dose-dependent manner. These results add to existing evidence that describes the role of proteasome inhibitors in inducing apoptosis in tumour cells. More importantly, these data emphasize the dependence of MB tumours on UPP activity.

We also reported that NPI-0052 induced mitochondrial hyperpolarization with significant ROS production leading to reduced GSH levels in MB cells. These data are in line with previous reports indicating that NPI-0052 induces oxidative stress followed by apoptosis activation in leukaemia cells^14^.

The p53 family comprises three members: p53, p63 and p73^27^. The members of this family are involved in apoptosis activation after DNA damage or oxidative stress^28^. Numerous evidence suggests that G3-MB and G4-MB normally express wild-type p53^3^. On the other hand, p73, a member of the p53 family, is overexpressed in MB tumours^6,29,30^. Importantly, p53 and p73 levels are regulated at post-transcriptional levels by fast degradation in the proteasome^31^. Therefore, we sought to assess whether NPI-0052 induces changes in p53 and p73 levels in MB cells. Overall, our study shows that NPI-0052 induces p53 and p73 stabilization in MB cells.

These data suggest that the cytotoxic effect of NPI-0052 in MB cells, which is mediated by the p53-family members’ stabilization, could be induced by two parallel mechanisms. A direct mechanism, mediated by NPI-0052 blocking the 26S proteasome complex and leading to increased levels of the p53 family. Second, an indirect mechanism, activated after NPI-0052-ROS generation causing DNA damage and followed by upregulation of p53, with subsequent apoptosis.

One of the principal roles of p53 is to assess the quality of cellular DNA as cells progress through the cell cycle^32^. If DNA damage is detected, p53 induces cell cycle arrest. It then acts on downstream target genes involved in DNA repair, or induces apoptosis through proteins such as Puma and BAX^33^. We show that increased concentrations of NPI-0052 induce a decrease in cell number in the S phase of the cell cycle with increased cell numbers in the G0/G1 or G2 phase. Importantly, both of these checkpoints are highly dependent on p53 activity^34^.

We verified the role of NPI-0052 in inducing p53 stabilization by measuring its expression in tumour organoids derived from G3/G4-MB cells that retain classical MB morphology. We observed similar results to those obtained in cell culture, specifically that NPI-0052 treatment leads to dose-dependent p53 stabilization with accumulation of γ-H_2_AX.

Another interesting finding from our study was that NPI-0052 has a synergistic apoptotic effect with γ-radiation. Radiation therapy is an integral part of metastatic MB treatment and is the source of the majority of treatment-related side effects^2^. Importantly, G3-MBs are often metastatic at diagnosis, necessitating radiation therapy. We first confirmed that G3/G4-MB primary cells and tumour organoids are sensitive to γ-radiation. A single dose of 2 Gy γ-radiation was sufficient to induce apoptosis in primary cells and tumour organoids. Next, we showed that 2Gy γ-radiation followed by NPI-0052 treatment has a synergistic effect resulting in a significant increase in apoptosis in primary G3/G4-MB cells and tumour organoids. Vlashi et al.^35^ reported that NPI-0052 induced radiosensitization in glioma tumours harbouring p53 mutant only, while here we suggest that radiosensitization is possible in G3/G4-MB with p53 wild type. Further experiments need to be performed to validate these results.

In summary, we show that NPI-0052 is an effective in vitro agent against human G3/G4-MB cells. We demonstrate that its cytotoxic effects are mediated through ROS generation, p53 stabilization and caspase activation leading to apoptosis. We also demonstrate that pre-treatment with γ-radiation sensitizes G3/G4-MB cells to NPI-0052 and increases cell death. Importantly, we were able to validate our cell culture results in a three-dimensional tumour organoid culture. These findings suggest that the proteasome may be a suitable target in the most aggressive form of MB, and raises the possibility that it could be implemented into MB treatment as an adjunct to radiation therapy, with the aim of maintaining survival while minimizing radiation-associated side effects.

## Supplementary information

Figure S1, Figure S2, Figure S3, Figure S4

Supplementary Figure Legend
