# Supplementary material for: NPI-0052 and γ-radiation induce a synergistic apoptotic effect in medulloblastoma
Source: Cell Death Dis. 2019 Oct 16;10(11):785. doi: 10.1038/s41419-019-2026-y (PMC6795856; doi:10.1038/s41419-019-2026-y)
Supplement: Supplementary file 1 — Figure S1, Figure S2, Figure S3, Figure S4 [file 41419_2019_2026_MOESM1_ESM.pptx]

## Slide 1
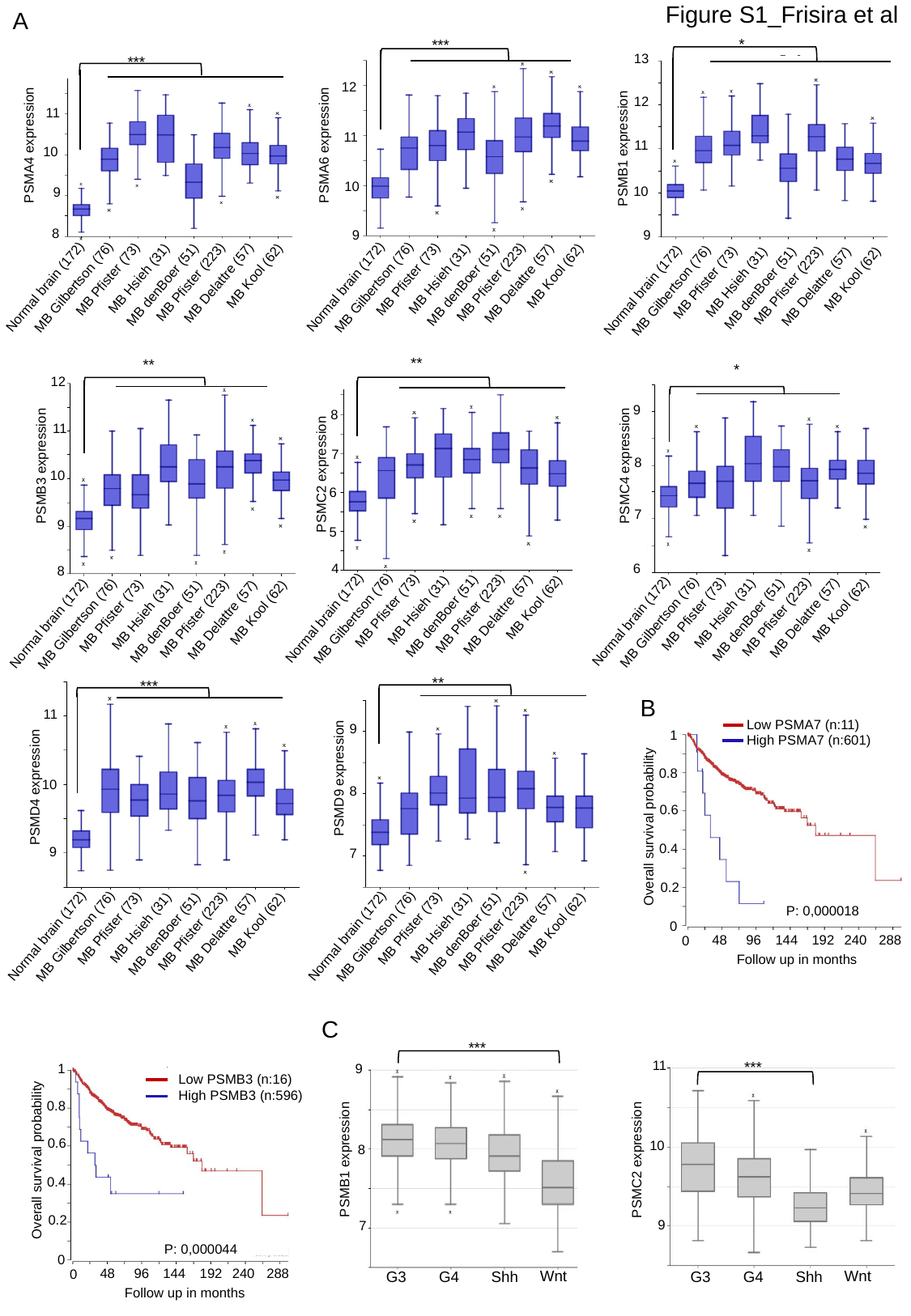

Figure S1_Frisira et al
***
PSMA6 expression
***
12
11
11
10
10
9
8
9
PSMA4 expression
MB Kool (62)
MB Hsieh (31)
MB Pfister (73)
MB Pfister (223)
MB Delattre (57)
MB denBoer (51)
Normal brain (172)
MB Gilbertson (76)
A
*
13
12
11
10
9
PSMB1 expression
MB Kool (62)
MB Hsieh (31)
MB Pfister (73)
MB Pfister (223)
MB Delattre (57)
MB denBoer (51)
Normal brain (172)
MB Gilbertson (76)
MB Kool (62)
MB Hsieh (31)
MB Pfister (73)
MB Pfister (223)
MB Delattre (57)
MB denBoer (51)
Normal brain (172)
MB Gilbertson (76)
*
9
PSMC4 expression
8
7
6
MB Kool (62)
MB Hsieh (31)
MB Pfister (73)
MB Pfister (223)
MB Delattre (57)
MB denBoer (51)
Normal brain (172)
MB Gilbertson (76)
PSMB3 expression
**
12
11
10
9
8
**
8
7
PSMC2 expression
6
MB Kool (62)
MB Hsieh (31)
MB Pfister (73)
MB Pfister (223)
MB Delattre (57)
MB denBoer (51)
Normal brain (172)
MB Gilbertson (76)
5
4
MB Kool (62)
MB Hsieh (31)
MB Pfister (73)
MB Pfister (223)
MB Delattre (57)
MB denBoer (51)
Normal brain (172)
MB Gilbertson (76)
**
PSMD9 expression
MB Kool (62)
MB Hsieh (31)
MB Pfister (73)
MB Pfister (223)
MB Delattre (57)
MB denBoer (51)
Normal brain (172)
MB Gilbertson (76)
9
8
7
***
MB Kool (62)
MB Hsieh (31)
MB Pfister (73)
MB Pfister (223)
MB Delattre (57)
MB denBoer (51)
Normal brain (172)
MB Gilbertson (76)
11
PSMD4 expression
10
9
B
Low PSMA7 (n:11)
High PSMA7 (n:601)
1
0.8
0.6
Overall survival probability
0.4
0.2
0
288
240
192
96
48
144
0
Follow up in months
P: 0,000018
C
***
9
PSMB1 expression
8
7
Wnt
Shh
G3
G4
11
10
PSMC2 expression
9
Wnt
Shh
G3
G4
***
1
0.8
0.6
Overall survival probability
0.4
0.2
0
288
240
192
96
48
144
0
Follow up in months
Low PSMB3 (n:16)
High PSMB3 (n:596)
P: 0,000044

## Slide 2
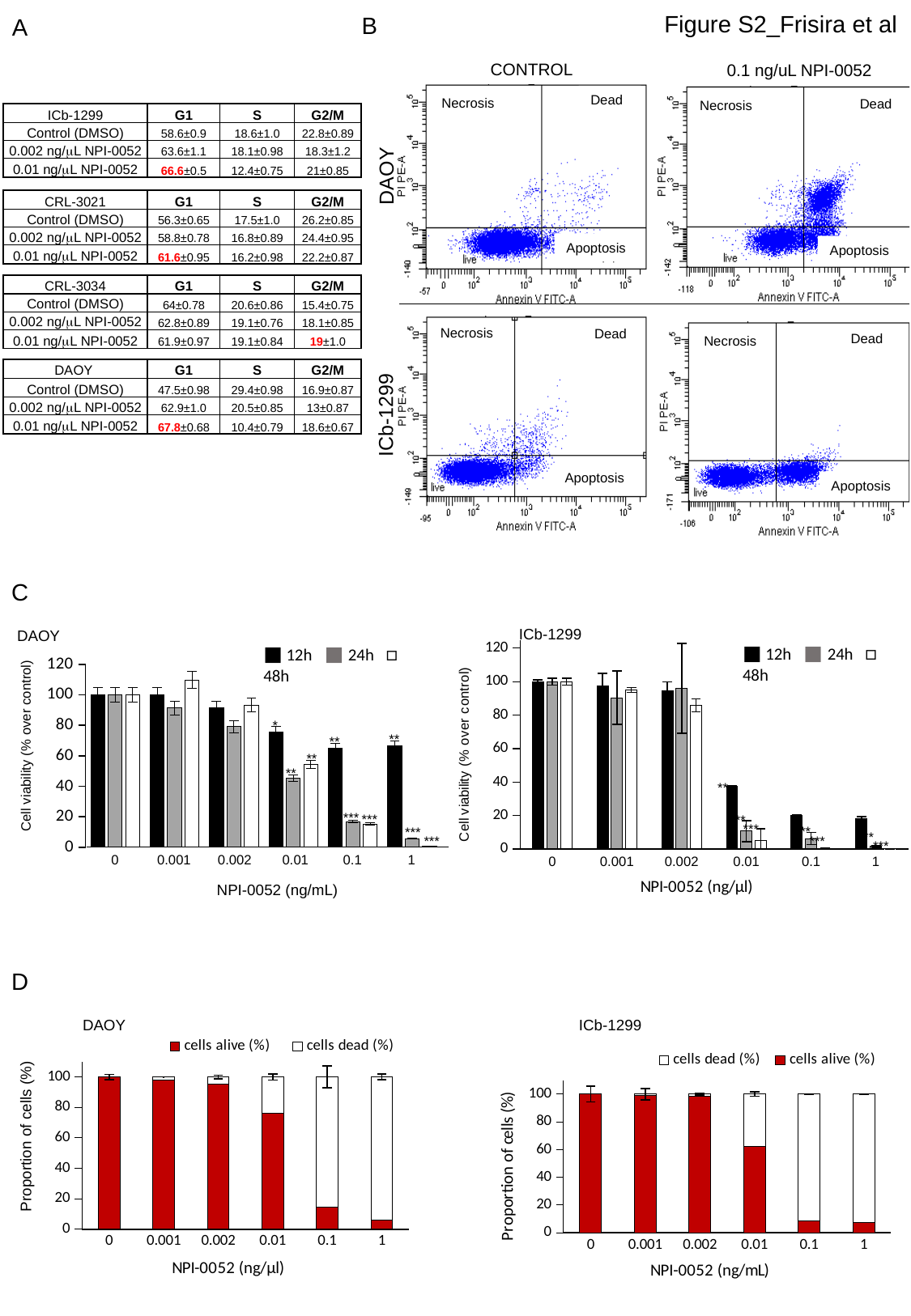

Figure S2_Frisira et al
B
A
CONTROL
0.1 ng/uL NPI-0052
Dead
Necrosis
Dead
Necrosis
| ICb-1299 | G1 | S | G2/M |
| --- | --- | --- | --- |
| Control (DMSO) | 58.6±0.9 | 18.6±1.0 | 22.8±0.89 |
| 0.002 ng/mL NPI-0052 | 63.6±1.1 | 18.1±0.98 | 18.3±1.2 |
| 0.01 ng/mL NPI-0052 | 66.6±0.5 | 12.4±0.75 | 21±0.85 |
| | | | |
| CRL-3021 | G1 | S | G2/M |
| Control (DMSO) | 56.3±0.65 | 17.5±1.0 | 26.2±0.85 |
| 0.002 ng/mL NPI-0052 | 58.8±0.78 | 16.8±0.89 | 24.4±0.95 |
| 0.01 ng/mL NPI-0052 | 61.6±0.95 | 16.2±0.98 | 22.2±0.87 |
| | | | |
| CRL-3034 | G1 | S | G2/M |
| Control (DMSO) | 64±0.78 | 20.6±0.86 | 15.4±0.75 |
| 0.002 ng/mL NPI-0052 | 62.8±0.89 | 19.1±0.76 | 18.1±0.85 |
| 0.01 ng/mL NPI-0052 | 61.9±0.97 | 19.1±0.84 | 19±1.0 |
| | | | |
| DAOY | G1 | S | G2/M |
| Control (DMSO) | 47.5±0.98 | 29.4±0.98 | 16.9±0.87 |
| 0.002 ng/mL NPI-0052 | 62.9±1.0 | 20.5±0.85 | 13±0.87 |
| 0.01 ng/mL NPI-0052 | 67.8±0.68 | 10.4±0.79 | 18.6±0.67 |
DAOY
Apoptosis
Apoptosis
Necrosis
Dead
Dead
Necrosis
ICb-1299
Apoptosis
Apoptosis
C
ICb-1299
DAOY
■ 12h ■ 24h □ 48h
### Chart
| Category | | | |
|---|---|---|---|
| control | 100.0 | 100.0 | 100.0 |
| NPI 0052 0.000000001 | 109.69 | 100.0 | 91.285 |
| NPI 0052 0.000000002 | 93.307 | 91.32 | 79.0727 |
| NPI 0052 0.00000001 | 54.36 | 75.66999999999999 | 45.3409 |
| NPI 0052 0.0000001 | 15.237999999999998 | 65.01 | 16.732099999999996 |
| NPI 0052 0.000001 | 0.4732 | 66.35 | 5.615889999999999 |0 0.001 0.002 0.01 0.1 1
### Chart
| Category | | | |
|---|---|---|---|
| 0 | 100.0 | 100.0 | 100.0 |
| 1E-3 | 97.48697093932883 | 90.37318330336332 | 95.0 |
| 2E-3 | 94.60027825186175 | 95.99478121306333 | 85.86805626851907 |
| 0.01 | 37.62592162410296 | 10.706781811883445 | 5.152174437051447 |
| 0.1 | 20.00492636256276 | 6.2481830857687966 | 0.3512095481859679 |
| 1 | 18.035549644785892 | 1.4919150580629215 | 0.10436297962109721 |■ 12h ■ 24h □ 48h
Cell viability (% over control)
NPI-0052 (ng/mL)
*
**
**
**
**
**
***
***
***
***
***
***
***
***
***
***
D
ICb-1299
DAOY
### Chart
| Category | | |
|---|---|---|
| 0 | 100.0 | 0.0 |
| 1E-3 | 97.89416846652269 | 2.105831533477314 |
| 2E-3 | 95.41036717062615 | 4.589632829373641 |
| 0.01 | 76.34989200863905 | 23.6501079913607 |
| 0.1 | 14.57883369330454 | 85.42116630669547 |
| 1 | 5.831533477321814 | 94.16846652267795 |Proportion of cells (%)
### Chart
| Category | | |
|---|---|---|
| 0 | 100.0 | 0.0 |
| 1E-3 | 98.8404196576477 | 1.159580342352285 |
| 2E-3 | 98.39867476532302 | 1.601325234676977 |
| 0.01 | 62.00993926007731 | 37.99006073992269 |
| 0.1 | 8.33793484262839 | 91.66206515737127 |
| 1 | 7.620099392600773 | 92.37990060739921 |

## Slide 3
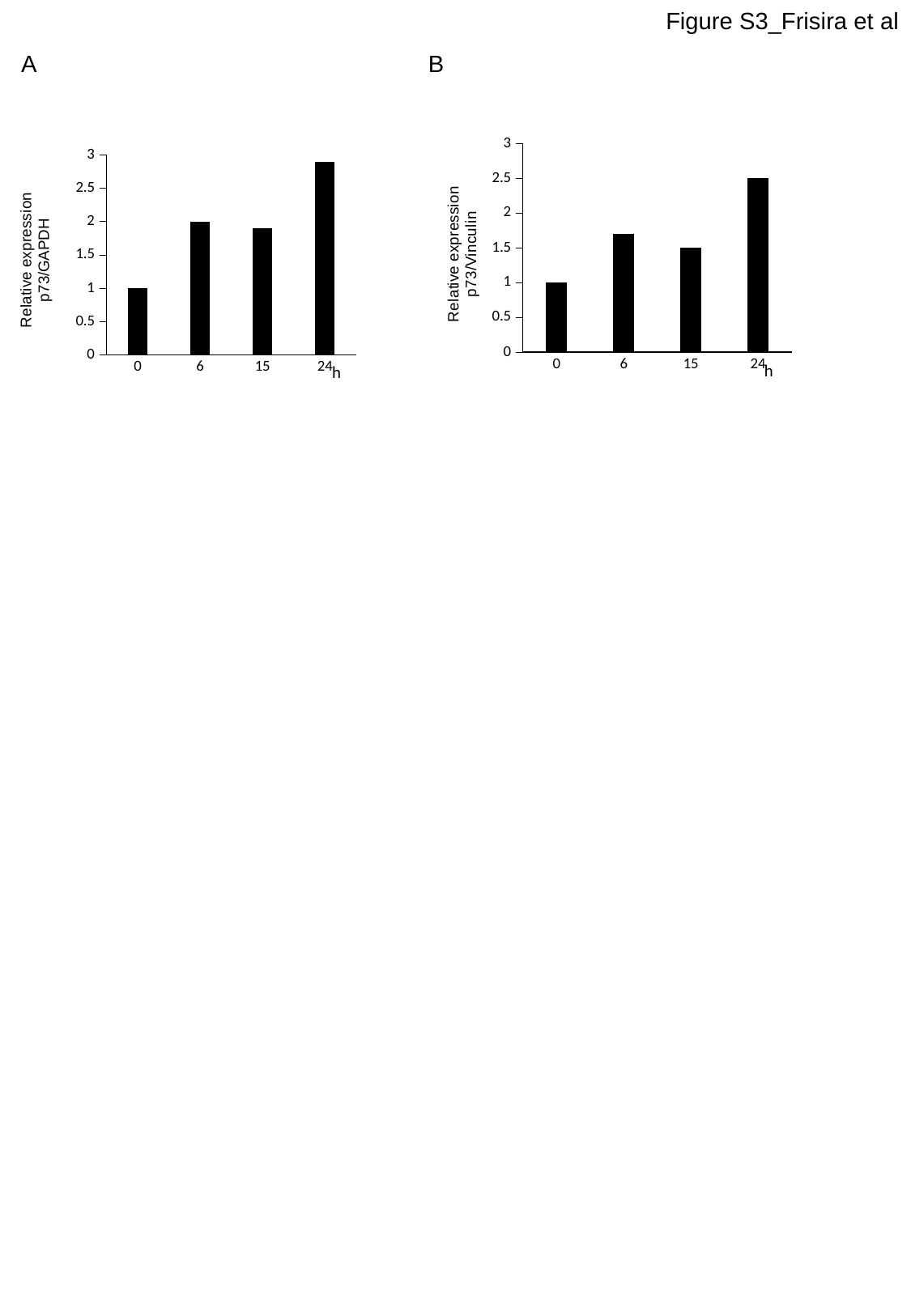

Figure S3_Frisira et al
A
B
### Chart
| Category | |
|---|---|
| 0 | 1.0 |
| 6 | 1.7 |
| 15 | 1.5 |
| 24 | 2.5 |
### Chart
| Category | |
|---|---|
| 0 | 1.0 |
| 6 | 2.0 |
| 15 | 1.9 |
| 24 | 2.9 |

## Slide 4
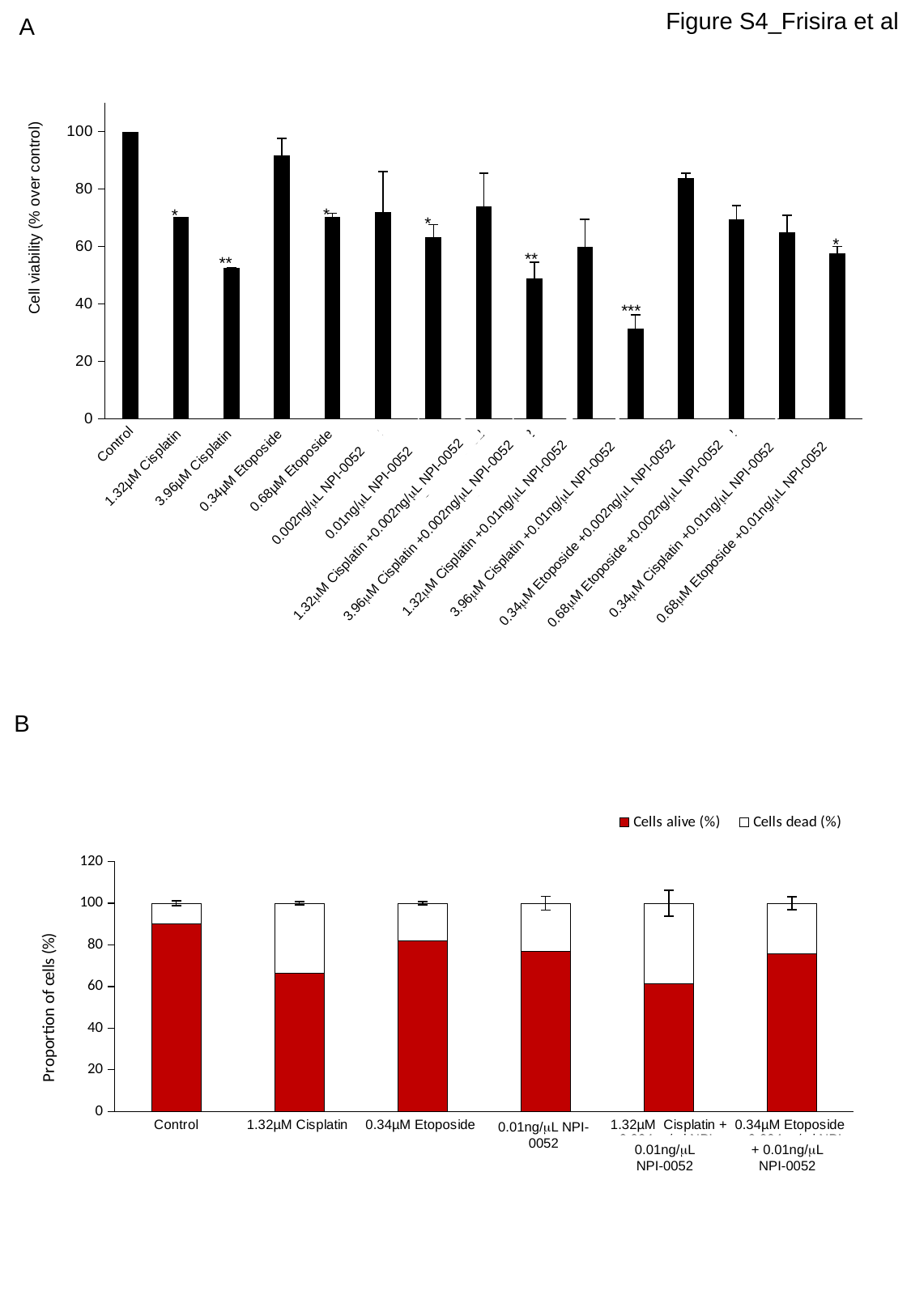

Figure S4_Frisira et al
A
### Chart
| Category | |
|---|---|
| Control | 100.0 |
| 1.32µM Cisplatin | 70.17639405265999 |
| 3.96µM Cisplatin | 52.63581315738071 |
| 0.34µM Etoposide | 91.61681305474903 |
| 0.68µM Etoposide | 70.33116923963172 |
| 0.002ng/ml NPI-0052 | 72.04677355810773 |
| 0.004ng/ml NPI-0052 | 63.28864992997871 |
| 1.32µM Cisplatin + 0.002ng/ml NPI-0052 | 73.85971243266866 |
| 3.96µM Cisplatin + 0.002ng/ml NPI-0052 | 48.826688208282704 |
| 1.32µM Cisplatin + 0.004ng/ml NPI-0052 | 59.80380796620438 |
| 3.96µM Cisplatin + 0.004ng/ml NPI-0052 | 31.40065750485845 |
| 0.34µM Etoposide + 0.002ng/ml NPI-0052 | 83.88086780047078 |
| 0.68µM Etoposide + 0.002ng/ml NPI-0052 | 69.55183065111521 |
| 0.34µM Etoposide + 0.004ng/ml NPI-0052 | 64.93489510049628 |
| 0.68µM Etoposide + 0.004ng/ml NPI-0052 | 57.61609794372473 |Cell viability (% over control)
0.01ng/mL NPI-0052
0.002ng/mL NPI-0052
1.32mM Cisplatin +0.01ng/mL NPI-0052
1.32mM Cisplatin +0.002ng/mL NPI-0052
3.96mM Cisplatin +0.01ng/mL NPI-0052
0.34mM Cisplatin +0.01ng/mL NPI-0052
3.96mM Cisplatin +0.002ng/mL NPI-0052
0.34mM Etoposide +0.002ng/mL NPI-0052
0.68mM Etoposide +0.01ng/mL NPI-0052
0.68mM Etoposide +0.002ng/mL NPI-0052
*
*
*
*
**
**
***
B
### Chart
| Category | Cells alive (%) | Cells dead (%) |
|---|---|---|
| Control | 90.05000000000001 | 9.949999999999996 |
| 1.32µM Cisplatin | 66.6 | 33.400000000000006 |
| 0.34µM Etoposide | 81.9 | 18.1 |
| 0.004ng/ml NPI-0052 | 76.8 | 23.200000000000003 |
| 1.32µM Cisplatin + 0.004ng/ml NPI-0052 | 61.25 | 38.75 |
| 0.34µM Etoposide + 0.004ng/ml NPI-0052 | 75.6 | 24.4 |0.01ng/mL NPI-0052
0.01ng/mL NPI-0052
+ 0.01ng/mL NPI-0052
