# Supplementary material for: NPI-0052 and γ-radiation induce a synergistic apoptotic effect in medulloblastoma
Source: Cell Death Dis. 2019 Oct 16;10(11):785. doi: 10.1038/s41419-019-2026-y (PMC6795856; doi:10.1038/s41419-019-2026-y)
Supplement: Supplementary file 2 — Supplementary Figure Legend [file 41419_2019_2026_MOESM2_ESM.docx]

**Supplementary Figure Legend:**

**Figure S1.** **26S overexpression indicates poor prognosis in MBs patient.** A) Expression of PSMA4, PSMA6, PSMB1, PSMB3, PSMC2, PSMC4, PSMD4 and PSMD9 in normal brain (172) versus human MB tumours. The data are derived from a previously published expression array. [R2: Genomics Analysis and Visualization Platform (https://hgserver1.amc.nl/cgi-bin/r2/main.cgi)]; significance was calculated with a one-way ANOVA between groups. B) Kaplan-Meier survival curve based on high and low PSMA7 and PSMB3 expression levels in MB tumours derived from Cavalli cohort with 763 patients. C) PSMB1 and PSMC2 levels in the four different groups: Group 3, Group 4, WNT and SHH. The data are derived from a previously published expression array (***P=0.0001).

Data were acquired from the R2: Genomics Analysis and Visualization Platform (https://hgserver1.amc.nl/cgi-bin/r2/main.cgi) and significance is calculated with a one-way ANOVA between groups.

**Figure S2. NPI-0052 inhibits proteasome activity and induce cell death of medulloblastoma.** A) Effect of different concentrations of NPI-0052 in cell cycle progression in human MB cells (ICb-1299, CHLA-01-MED, CHLA-01R-MED and DAOY). MBs cells were treated with NPI-0052 (0, 0.002, and 0.1 ng/μL) for 24 hrs and then fixed with 70% ethanol, washed and RNAse treated and labelled with 50 μg/mL PI. Cell cycle analysis of treated and untreated cells was performed. Data are presented as the relative fluorescence intensity of cell sub-populations in the 2-dimensional FACS profile or in percentage of cells in a given sub-population. B) DAOY and ICb-1299 cells were treated or not with 0.1 ng/μL NPI-0052 for 24 hours. MB cells were labelled with Annexin V-FITC and PI. Cells that stain negative for Annexin V-FITC and negative for PI were consider as “alive”. Dead cells were considered to be the apoptotic, necrotic and dead cells. C) DAOY and ICb-1299 cells were treated or not with different concentrations of NPI-0052 for 12, 24 and 48 hours. Cell viability were determined with CellTiter-Glo. (n=4) ± SEM. (*) P < 0.01; (**) P < 0.001; (***) P < 0.0001. D) DAOY and ICb-1299 cells were treated or not with different concentrations of NPI-0052. After 48 hours, cells were collected and apoptosis was measured with Annexin V-FITC and PI for flow cytometry analysis. (n=3). Data are represented as mean ± SD.

**Figure S3. NPI-0052 induced stabilization of the p53-family.** Human medulloblastoma cells DAOY and ICb-1299 were treated with 0.002 ng/μL NPI-0052 for 0, 6, 15 and 24 hours. Cells were collected and a western blot of lysed cells probed with a primary anti-p73 (1/1000), anti-p53 (1/1000), GAPDH (1/5000) or vinculin (1/5000) antibodies. A-B) Western blot bands for p73 were quantitated by ImageJ in DAOY (A) and ICb-1299 (B).

**Extended Material and Methods**

**Cell culture**

Medulloblastoma cells, DAOY and ICb-1299, were cultured at 37°C in humidified 5% CO_2_ in DMEM + GlutaMAX medium (Gibco), supplemented with 10% v/v foetal bovine serum (FBS, Gibco) and penicillin/streptomycin (1 U/ml, Gibco), as previously described^1^.

CHLA-01-MED (ATCC®CRL-3021) and CHLA-01R-MED (ATCC®CRL-3034) were cultured 37°C in humidified 5% CO2 in DMEM:F12 Medium (Gibco) with 20 ng/mL human EGF, 20 ng/mL human basic FGF and 2% (v/v) B-27 Supplement (Invitrogen, Cat. No.17504).

DAOY cells were purchased from ATCC (ATCC® HTB186™). The primary human MB cells, ICb-1299, were obtained from Dr Xiao-Nan Li, Baylor College of Medicine, Texas Children’s Cancer Centre, USA. The human primary MB cells CHLA-01-MED and CHLA-01R-MED were donated by Prof Silvia Marino, Queen Mary University of London, UK.

**Isolation and culture of post-natal mouse cerebellar cells**

Cerebellar granule cells were isolated from postnatal day 7 C57BL/6 mice, using an enzymatic protocol, as described elsewhere^2^. Cells were plated at a density of 2.8 × 10^5^/well in 24-well plates treated with Poly-D-Lysine. Cultures were maintained in supplemented Basal Medium Eagle culture medium containing 10% horse serum, 32mM glucose, 2mM glutamine, antibiotic/antimycotic (Gibco®, Thermo Fisher Scientific, New York, NY, USA), and 25mM KCl, at 37°C. After 24 hours, 10μM cytosine arabinoside (AraC) was added to the culture to inhibit proliferation of non-neuronal cells.

**NPI-0052 preparation**

NPI-0052 (AdipoGen®) was dissolved in DMSO. All controls for each experiment were treated with DMSO with equal volumes as the drug treatment for the rest of the conditions.

**Cell counting**

MB cells were counted using a NucleoCounter® NC-100™ (Chemometec) according to manufacturer’s instructions.

**Cell Irradiation**

Cells and organoids were irradiated with gamma radiation using a caesium (137Cs) irradiator at the dose rate of 2 or 4 gray (Gy) per minute at UCL-Institute of Child Health^3^.

**Knockdown of p73**

siRNA constructs targeting p73 (sip73*1: ID: 2671, sip73*2: ID: 115666) and a non-targeting control siRNA (scramble) were purchased from Ambion. Cells were transfected with 10 pM siRNA with lipofectamine 3000 (Life Technologies) according to the supplier’s protocol. Cells were collected at different time points after transfection to assess knockdown efficiency.

**Cell cycle progression**

Cells were seeded and treated as indicated. After 24 hours, MB cells were trypsinized, harvested and fixed in 1 ml 70% cold ethanol in FACS tubes and incubated at 4°C for 15 min. After incubation, cells were centrifuged at 1,500 rpm for 5 min and the cell pellets were resuspended in 500μl propidium iodine (10μg/ml) containing 300μg/ml RNase (Sigma, MO, USA). Then, cells were incubated on ice for 30 min and filtered with 53μm nylon mesh. Cell cycle distribution was calculated from 10,000 cells using a BD FACS CANTO II flow cytometer.

**Apoptosis determination**

Apoptosis was quantified using the Annexin V-FITC Determination Kit (eBioscience) according to the manufacturer’s instructions. Briefly, cell medium and cell pellet were collected in a FACS tube. Annexin V-FITC (25μg/ml) was added to the cells. The cells were incubated for 10 minutes in darkness at room temperature. 1μg/ml propidium iodide staining solution was then added and FACS was carried out using a BD FACS CANTO II flow cytometer.

**Real-time PCR**

Total RNA was extracted from samples using Rneasy Mini Kit (Qiagen, Hilden, Germany) according to the manufacturer’s instructions. RNA was quantified using spectrophotometry. DNA in the sample was then digested using Dnase 1 (Sigma) and RNA was converted to cDNA using RevertAid H minus First Strand cDNA Synthesis Kit (Thermo Fisher Scientific). For real-time PCR, we used SYBR green Supermix (Bio-Rad Laboratories, Hercules, CA, USA) according to the manufacturer’s instructions.

Forward and reverse human primers used were (written 5’ to 3’):

TAp73 (fwd: GCACCTACTTTGACCTCCCC, rev: GCACTGCTGAGCAAATTGAAC);

TP53 (fwd: CGTCTGGGCTTCTTGCATTC, rev: AAGACCTGCCCTGTGCAGC);

18S (fwd: CAGCCACCGAGATTGAGCA, rev: TAGTAGCGACGGGCGGTGTG);

**Protein quantification**

Protein quantification was perform using the Pierce™ Assay Kit according to the manufacturer’s instructions. Absorbance was measured at 595nm which was carried out using a plate reader. We used the bovine serum albumin (BSA) solutions as a standard.

**Western blotting**

After treatment, MB cells were collected and sonicated for 5 min in RIPA buffer (50 mM Tris–HCl (pH 7.5), 150 NaCl, 1% NP-40, 0.25% sodium deoxycholate). Proteins were separated by SDS-PAGE. Proteins were transferred onto nitrocellulose membranes, which were blocked in 5% non-fat milk diluted in Tris buffered saline (TBS) containing 0.5% Tween 20 (TBS-T) for 30 min at room temperature. Primary antibodies were diluted in 5% milk in TBS-T and incubated at 4°C overnight.

We used rabbit p73 (1/1000, Bethyl), mouse ubiquitin (1/1000, Cell Signalling), mouse p53 (1/1000, Santa Cruz), rabbit caspase 8 (1/1000, Cell Signalling) and rabbit caspase 9 (1/1000, Cell Signalling). Mouse GAPDH (1/5000, Sigma Aldrich) and mouse vinculin (1/5000, Cell Signalling) were used as a loading control.

The membranes were then washed three times for 10 min in TBS-T. Secondary antibodies, anti-mouse (1/10000, Sigma-Aldrich) or anti-rabbit (1/10000, Sigma-Aldrich) were diluted in 5% non-fat milk in TBS-T and incubated for 1 hour at room temperature. The membranes were washed in TBS-T three times for 10 min. Then, membranes were incubated in Lumi-Light Western blotting substrate (Roche Diagnostics, Indianapolis, IN). Membranes were exposed to film and developed.

**Cell viability**

Cell viability was quantified by CellTiter-Glo® Luminescent Cell Viability Assay according to the manufacturer’s instructions. Detection is based on using the luciferase reaction to measure the amount of ATP from viable cells. Luminescence was measured using a Synergy HT Multi-Mode Microplate Reader.

**Measurement of total Glutathione and GSH/GSSG levels**

MB cells were plated and treated as indicated. Reduced and oxidized glutathione ratio was measured by using the GSH/GSSG-Glo assay kit (Promega) according to the manufacturer's protocol. Assay reagents were added directly to cells cultured in multi well plates. The GSH/GSSG-Glo™ Assay is a luminescence-based system for the detection and quantification of GSH/GSSG ratios in cultured cells. The luminescence intensity was measured using a Synergy HT Multi-Mode Microplate Reader.

**Mitochondrial membrane potential**

3,3’-dihexyloxacarbocyanine iodide (DiOC6) (Invitrogen, Cat. No.D273) staining was carried out as previously described^4^. Briefly, MB cells were washed once in FACS buffer and then resuspended in 300μL of the same buffer containing 4nM DiOC6. Cells were then incubated for 30 min at RT, followed by one wash with PBS. Cells were resuspended in PBS containing 200ng/mL DAPI staining.

**Hydrogen Peroxide Assay**

Amplex Red Hydrogen Peroxide/Peroxidase Assay Kit (ThermoFisher Scientific) was used to determine the amount of Hydrogen Peroxide (H2O2) present in cell samples as a marker for oxidative stress. Absorbance at 562nm was measured using a Synergy HT Multi-Mode Microplate Reader.

**Tumour organoid**

Tumour organoids were grown as previously described^5^ with some modifications. 200μl of tumour cell suspension (1x10^6^ cells) was mixed with 800μl of cold matrigel (Life Sciences). 20μl of this suspension was pipetted onto a dimpled parafilm mold. Once the matrigel droplets had set after 15 minutes incubation at 37⁰C, they were transferred to a 10cm tissue culture dish and suspended in 10mL of the appropriate medium. Tumour organoids were incubated at 37⁰C in humidified CO_2_ without shaking for 4 days. Then, the tumour organoids were transferred to an orbital shaker and cultured at 70 revolutions per minute (RPM). Medium was changed twice weekly.

**Immunostaining**

Paraffin embedded tumour organoids were sectioned at 3-μm and staining for hematoxylin and eosin (H&E), p53 (Dako, prediluted), cleaved caspase 3 (Novocastra, prediluted) and gamma-H_2_AX (Roche, prediluted), were performed by UCL IQPath (Institute of Neurology, London, UK). Immunostaining was done on Ventana Discovery XT instrument, an automated staining machine (ROCHE, Burgess Hill, UK) following the manufacturer's guidelines, using horseradish-peroxidase-conjugated streptavidin complex and diaminobenzidine as a chromogen.

**Image capturing and analysis**

Histological slides were digitised on a LEICA SCN400 scanner (LEICA UK) at 40×magnification and 65% image compression setting. Images were stored on Slidepath Digital Image Hub (Leica Microsystems).

**Medulloblastoma patient tumour data**

The R2 platform (https://hgserver1.amc.nl/cgi-bin/r2/main.cgi?&species=hs) is a web server for investigating the RNA sequencing and microarray data of expression, co-expression, and survival data of normal and tumour samples from the TCGA, GEO, and GTEx projects. Kaplan-Meier analysis and subgroup expression analysis was performed using the Cavalli data set.

**REFERENCES**

1 Merve, A. *et al.* Polycomb group gene BMI1 controls invasion of medulloblastoma cells and inhibits BMP-regulated cell adhesion. *Acta Neuropathol Commun* **2**, 10, doi:10.1186/2051-5960-2-10 (2014).

2 Spataru, A., Le Duc, D., Zagrean, L. & Zagrean, A. M. Ethanol exposed maturing rat cerebellar granule cells show impaired energy metabolism and increased cell death after oxygen-glucose deprivation. *Neural Regen Res* **14**, 485-490, doi:10.4103/1673-5374.245474 (2019).

3 Blattner, C., Tobiasch, E., Litfen, M., Rahmsdorf, H. J. & Herrlich, P. DNA damage induced p53 stabilization: no indication for an involvement of p53 phosphorylation. *Oncogene* **18**, 1723-1732, doi:10.1038/sj.onc.1202480 (1999).

4 Niklison-Chirou, M. V. *et al.* TAp73 is a marker of glutamine addiction in medulloblastoma. *Genes Dev* **31**, 1738-1753, doi:10.1101/gad.302349.117 (2017).

5 Hubert, C. G. *et al.* A Three-Dimensional Organoid Culture System Derived from Human Glioblastomas Recapitulates the Hypoxic Gradients and Cancer Stem Cell Heterogeneity of Tumors Found In Vivo. *Cancer Res* **76**, 2465-2477, doi:10.1158/0008-5472.CAN-15-2402 (2016).
